# Rapid assay development for low input targeted proteomics using a versatile linear ion trap

**DOI:** 10.21203/rs.3.rs-4702746/v1

**Published:** 2024-07-19

**Authors:** Brian Searle, Ariana Shannon, Rachael Teodorescu, No-Joon Song, Lilian Heil, Cristina Jacob, Philip Remes, Zihai Li, Mark Rubinstein

**Affiliations:** Ohio State University; Ohio State University; Ohio State University; The Ohio State University; Thermo Fisher Scientific; Thermo Fisher Scientific; Thermo Fisher Scientific; The Ohio State University Comprehensive Cancer Center – James Cancer Hospital and Solove Research Institute; The Ohio State University

## Abstract

Advances in proteomics and mass spectrometry enable the study of limited cell populations, where high-mass accuracy instruments are typically required. While triple quadrupoles offer fast and sensitive low-mass accuracy measurements, these instruments are effectively restricted to targeted proteomics. Linear ion traps (LITs) offer a versatile, cost-effective alternative capable of both targeted and global proteomics. Here, we describe a workflow using a new hybrid quadrupole-LIT instrument that rapidly develops targeted proteomics assays from global data-independent acquisition (DIA) measurements without needing high-mass accuracy. Using an automated software approach for scheduling parallel reaction monitoring assays (PRM), we show consistent quantification across three orders of magnitude in a matched-matrix background. We demonstrate measuring low-level proteins such as transcription factors and cytokines with quantitative linearity below two orders of magnitude in a 1 ng background proteome without requiring stable isotope-labeled standards. From a 1 ng sample, we found clear consistency between proteins in subsets of CD4^+^ and CD8^+^ T cells measured using high dimensional flow cytometry and LIT-based proteomics. Based on these results, we believe hybrid quadrupole-LIT instruments represent an economical solution to democratizing mass spectrometry in a wide variety of laboratory settings.

## Introduction

Systems biology is the study of interactions within and between cells, where the goal is to learn how those interactions give rise to the complex behavior seen in an entire system.^1^ One challenge is that many complex biological processes, such as adaptive immunity, are built from small populations of distinct cell types acting in concert.^2,3^ Improvements in proteomics methods and mass spectrometry (MS) instrumentation have paved the way for low-input and single-cell proteomics, which make it possible to study how limited cell populations contribute to the whole. While the majority of single-cell methods use tandem mass tags (TMT)^4^ to increase signal (and thus consistency) with data-dependent acquisition (DDA),^5,6^ several groups have demonstrated that data-independent acquisition (DIA) is an effective solution to measuring low-input samples.^7–9^ However, high-mass accuracy instruments are required in nearly all cases.

While single-cell and low-input global proteomics is typically acquired using high-mass accuracy instruments, nominal-mass instruments, such as triple quadrupoles, lead in quantitative sensitivity using targeted selected reaction monitoring (SRM).^10^ With SRM, peptides are detected based on monitoring multiple fragment ion signals produced by each selected precursor ion. Transitions (diagnostic precursor/fragment ion pairs) in a pre-specified schedule must be provided to the instrument for monitoring at specific times within the chromatographic gradient.^11^ While triple quadrupoles are extremely quick instruments capable of rapidly switching between ion pairs, they can only monitor a single *m/z* at a time. As such, triple quadrupoles are limited to targeted experiments, which require a high-mass resolution instrument to select and schedule targeted peptides and transitions before migrating to a nominal-mass instrument for high-throughput monitoring.

An alternative targeted method to SRM is parallel reaction monitoring (PRM), which uses a quadrupole-equipped high-resolution mass spectrometer where the third quadrupole is replaced with an Orbitrap^™^ (Q-Orbitrap, also known as a Q-Exactive^™^) or a time-of-flight analyzer (Q-ToF). Rather than measure precursor/fragment transitions, all precursor-specific fragment ions are collected in a full tandem mass spectrum with PRM.^12^ A major advantage of PRM is that diagnostic fragment ions are selected after the experiment is performed, which can vastly simplify the assay development process. PRM has provided meaningful biological insight into several diseases, including systemic autoimmune diseases,^13^ multiple sclerosis,^14^ and colorectal cancer.^15^ When coupled with global proteomics, PRM is a powerful tool for interrogating system-wide interactions between cells.

Linear ion traps (LITs) are another versatile, fast, nominal-mass analyzer comparable in resolution and complexity to triple quadrupoles. Modern Thermo Scientific^™^ Tribrid^™^ instruments have incorporated LITs as a tertiary analyzer, coupled with an Orbitrap.^16^ Using a Tribrid instrument, Heil et al^17^ showed that the benefit of PRM lies within its ability to monitor multiple product ions produced within a selected precursor *m/z* range and that the LIT in Tribrids was an effective readout for targeted proteomics. A LIT measures ions trapped in an electric field by adjusting RF and DC voltages to selectively eject ions based on their *m/z* to collect MS/MS spectra. Unlike triple quadrupoles, which have to “dwell” at each increment of m/z to form a spectrum, LITs acquire full scan MSn data quickly and sensitively,^18^ making them also viable for global proteomics using DDA or DIA.^19^ As a result, a hybrid quadrupole-LIT (Q-LIT) could act as an “all-in-one” nominal-mass instrument capable of both targeted and global proteomics.

As with triple quadrupoles, LITs are extremely sensitive, ion-efficient mass analyzers apt for low-input proteomics.^20^ In some circumstances, LITs can be more effective than high-resolution mass analyzers for low-input samples (≤ 10 ng)^21^ and can measure single cells without multiplexing reagents.^22^ At higher sample input (≥ 100 ng), the lack of high mass accuracy overshadows the increased sensitivity of LITs. There exist other compelling reasons to consider LIT-based instruments in high-throughput applications. In particular, LITs operate at high pressure (10^− 3^ mTorr) in comparison to ToF analyzers (10^− 6^ mTorr), where ions have to travel uninterrupted for meters, or Orbitrap analyzers (10^− 10^ mTorr), where ions can travel for more than a kilometer. Lower vacuum pump requirements allow LITs to be built more affordably, robustly, and housed in smaller instrument footprints.

Here, we present a workflow using a hybrid quadrupole-LIT (Q-LIT) instrument from Thermo Scientific as a single instrument for rapidly generating targeted assays for low-input experiments. With the Q-LIT, we demonstrate how to build nominal-mass targeted transition libraries using both DDA and gas-phase fractionated (GPF) DIA libraries. We then show the quantitative accuracy of targeted PRMs with a Q-LIT using matched-matrix calibration curves collected with 1, 10, and 100 ng total protein to model low abundant immune cell populations. To facilitate this, we developed an open-source software tool that directly schedule-optimized PRM assays from DDA and DIA libraries. Finally, we show quantitative consistency measuring low-level biological targets in cytokine-stimulated CD4^+^ and CD8^+^ T cells with as little as 1 ng on column. These results suggest that Q-LITs can perform as inexpensive stand-alone instruments for quantitative proteomics, capable of a wide range of measurements without needing high-resolution mass spectrometry.

## Results and Discussion

Linear ion traps (LITs) are robust, sensitive, and fast mass analyzers, yet these instruments have limited mass resolution. Previously, our lab demonstrated that LITs could be used effectively as stand-alone mass analyzers to measure low-input samples using an Orbitrap Eclipse^™^ Tribrid mass spectrometer.^22^ In that work, we detected approximately 400 proteins from single cells using data-independent acquisition coupled with chromatogram libraries to help make detections.^23^ While our Eclipse instrument configuration ignored the high-resolution Orbitrap mass analyzer, we performed those experiments in the context of a high-end Tribrid instrument. Furthermore, the 400 proteins we measured were the easiest to observe but not necessarily the most biologically useful to monitor. While reduced representation approaches^24,25^ that quantify a limited panel of easily observed proteins can help infer biological states, significant hurdles must be overcome to predict the expression patterns of unmeasured proteins. As such, directly measuring panels of proteins of interest in low-input samples using targeted proteomics may be preferable to global proteomics.

In this work, we sought to answer three remaining questions. First, by eliminating the Orbitrap, could an affordable quadrupole-LIT (Q-LIT) mass spectrometer perform at a high level as a stand-alone instrument for both library generation and targeted proteomics measurement? Second, can a Q-LIT mass spectrometer quantify peptides at and below the level of single cells? Third, can quantitative experiments measure low-level biologically relevant proteins like cytokines and transcription factors at or below 1 ng? To this end, we assessed several parameters of the Stellar^™^ mass spectrometer, a new hybrid Q-LIT design produced by Thermo Scientific. First, we tested proteome-wide library generation; then, we assessed quantitative linearity using targeted PRM experiments with 100, 10, and 1 ng sample inputs. Finally, we tested sensitivity and measurement consistency in a biological context.

### A Q-LIT workflow for generating PRM assays using DDA and DIA data

**Figure 1:**
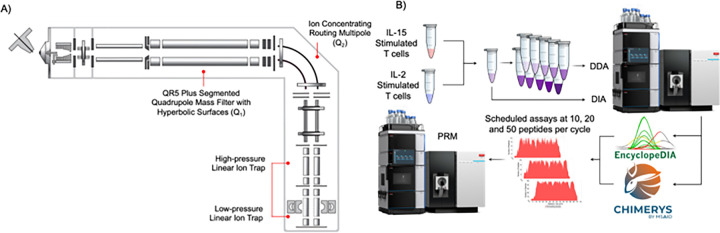
Overview of the instrument and workflow for developing targeted assays using a LIT. **A**) The instrument schematic of the Stellar MS. Ions enter the first QR5 Plus Segmented Quadrupole Mass Filter with Hyperbolic surface before entering into the Ion Concentrating Routing Multipole. The Ion Concentrating Routing Multipole behaves as the collision and storage cell. Ions are then moved to the high-pressure cell of the dual-pressure LIT, and eventually to the low-pressure cell for mass analysis. **B**) A schematic of the methodology taken. Chromatogram libraries were generated using a GPF-DIA approach, and DDA libraries were generated from offline high-pH reverse-phase fractionated proteomes. We searched samples using both EncyclopeDIA and CHIMERYS, where the combined results were used to schedule PRM assays at the 100, 10, and 1 ng levels using 50, 20, and 10 peptides per cycle, respectively.

The Stellar MS is a hybrid Q-LIT mass spectrometer with improved ion transmission features capable of performing rapid scans up to 200 kDa/s (Figure 1A). The instrument shares many of the same design components as existing Orbitrap-based instruments.^26–28^ Tribrid instruments accumulate fragment ions in the collision cell (also known as the ion routing multipole, IRM) before transfer to the mass analyzer. Analogously, the Stellar accumulates fragment ions in the collision cell (Q2, also known as the ion concentrating routing multipole, ICRM) before transfer to the LIT for mass analysis. These arrangements produce high scanning speeds by performing fragment accumulation in parallel with mass analysis in the low-pressure LIT.^29,30^

An advantage of the Q-LIT geometry is that it is suitable for both global discovery proteomics as well as targeted proteomics. To leverage this, we implemented a workflow to generate high-quality peptide libraries using off-line fractionated DDA or GPF-DIA, and software to build on-the-fly PRM assays for the same instrument (Figure 1B). Briefly, the software takes a DDA-based spectrum library or a DIA-based chromatogram library as input and compares it with potential targeted proteins. The target list contains a list of critical accession numbers along with other optionally desired entries in a selected FASTA database. The assay can be modified using both a peptide inclusion and exclusion list. Assays can be adjusted depending on instrument settings, where the maximum assay density and a retention time scheduling window width must be selected. A recent single-injection DIA run also ensures that the retention time schedule matches the current LC column conditions.

Peptides are selected using this software tool based on the highest signals recorded in the library. For DDA, this signal is based on precursor intensity if available. For GPF-DIA, this signal is based on the intensity of the third largest fragment ion per peptide following common SRM/PRM conventions of requiring at least three transitions.^29^ The algorithm chooses peptides using a greedy approach, where the most abundant peptides are scheduled first. After the algorithm chooses a specified number of peptides for a given protein (typically 3–5), no additional peptides from that protein are considered. Additionally, peptides cannot be added to a retention time region if any time point in that region has already reached the maximum assay density. Once the algorithm iterates through all possible peptides, the software tool produces a scheduling report and a target inclusion list for the Thermo method editor. While the tool focuses on simplifying scheduling for Thermo instruments, it is analyzer and vendor agnostic, supporting scheduling for both Orbitrap and ToF instruments. This software workflow has an accessible graphical user interface built into the EncyclopeDIA code base (see **Supplemental Note** for more details).

### Developing a comprehensive target library

PRM assays are commonly generated from various sources, including public repositories that store targeted proteomics data such as the PeptideAtlas,^31^ CPTAC,^32^ or Panorama.^33^ Additionally, assays can be built from global data, with targets selected from empirical measurements on the biological matrix of interest. For this work, we wanted to use methods that could be fully acquired on the Q-LIT but still be capable of detecting low-abundant peptides. One advantage of this approach is that targets are tuned for the instrument from the context of retention time scheduling and optimal transition selection.

**Figure 2:**
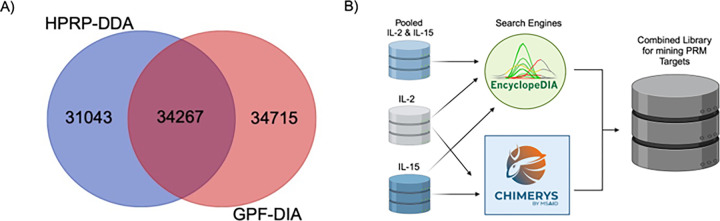
A spectral library using offline high-pH reverse phase fractionated data-dependent acquisition (HPRP-DDA) and the chromatogram library using gas-phase fractionated data-independent acquisition (GPF-DIA). **A**) A Venn diagram of the peptides detected from each library. **B**) Detections from CHIMERYS were combined with EncyclopeDIA to generate a combined search engine library to mine PRM targets.

To generate a low-input PRM assay on the Q-LIT, we tested two standard methods of building libraries: a chromatogram library using GPF-DIA and a spectral library using fractionated DDA. We collected libraries from a pool of IL-2 and IL-15-stimulated T cell proteomes. To build the chromatogram library, 6x gas-phase fractions were used with 2 *m/z* wide isolation windows across mass ranges of 100 *m/z* per injection. Since the background proteome matrix is not chemically altered or diluted, this approach produces retention times that closely match the quantitative PRM experiments. In contrast, we off-line fractionated the DDA library samples using high-pH reverse phase separations to yield a total of 10 fractions, which were analyzed in separate injections. Consequently, each fraction has a simplified matrix background, which may not reflect retention times as consistently in unfractionated quantitative samples. The DDA and DIA methods produced libraries that were similar in size but of surprisingly distinct populations of peptides (Figure 2A), presumably due to the different fractionation methods and matrix backgrounds used to generate each library.

In addition to producing slightly more peptide detections, peptide-centric extraction^34^ of DIA datasets is more akin to fragment-level quantification using targeted methods than DDA measurements.^35^ As such, peptides detected using GPF-DIA are more likely to produce robust, targeted assays since the mode of discovery uses similar methodologies to the final quantitative measurements. However, some sample types, such as enriched phosphopeptides, may be better suited to library generation with DDA since they can take advantage of stochastic sampling to detect more peptides and proteins over technical replicates.^36^ For this work, we chose to proceed with the GPF-DIA library for assay development, but the scheduling software produced for this work functions with either library source.

For DIA injections, search results from EncyclopeDIA and CHIMERYS were combined for downstream work (Figure 2B). CHIMERYS is a spectrum-centric search engine that builds on INFERYS to provide spectra and retention-time predictions for peptides in a given FASTA database.^37^ In comparison, we searched a Prosit-predicted spectral library^38,39^ with the peptide-centric search engine, EncyclopeDIA, which was adapted for analyzing ion trap data. Consequently, EncyclopeDIA was limited to searching +2 and +3 peptides to maintain a reasonable search space, while CHIMERYS was configured to consider modifications and higher charge states as well. More peptides were detected from CHIMERYS compared to EncyclopeDIA in each gas-phase fraction (**Supplemental Figure S1A**), but considering the superset of detections increased the total number of potential targets (**Supplemental Figure S1B**) and both search engines produced an equal number of viable peptide targets that could be used in downstream PRM experiments (**Supplemental Figure S1C**). In all cases, the retention times from CHIMERYS-detected peptides were re-peak picked using EncyclopeDIA to identify candidate target transitions for PRM measurement in a combined DIA library.

### Assessing Q-LIT PRM quantitative accuracy at low input

With low-input global proteomics, we preferentially measure only the most abundant proteins. We stress-tested the quantitative accuracy of the Q-LIT system using PRMs by measuring biologically relevant proteins that tend to occur at a range of levels in the proteome. To accomplish this, we first functionally annotated candidate peptides in the combined DIA library using the PANTHER database.^40^ We selected target proteins based on GO-terms and Reactome pathways for T cell differentiation, immune biology, T cell activation, cytokines, and transcription factors with a focus on choosing proteins associated with the dynamics of memory T cells. Using the PRM scheduling algorithm, we constructed three assays using the same bank of proteins, where each assay was suited to a different input level: up to 50 peptides/cycle for 100 ng of material, 20 peptides/cycle for 10 ng of material, and 10 peptides/cycle for 1 ng of material. Ultimately, the 100 ng assay monitored 481 peptides, the 10 ng assay monitored 151, and the 1 ng assay monitored 61. To maintain a 2-second cycle time using 1 ng of material, the maximum ion injection time (maxIIT) was set to 200 ms. Similarly, at 10 ng of material, the maxIIT was set to 95 ms (slightly below 100 ms to accommodate the additional time required to route ions in the mass spectrometer). At 100 ng of material, the ion injection time was set to 50 ms; however, each scan rarely met that length of time.

We performed matrix-matched calibration curves^41^ at 100 ng, 10 ng, and 1 ng levels to assess the quantitative accuracy of the Q-LIT over several orders of magnitude. Dilutions in a buffer background are useful to assess instrument sensitivity, but because background noise decreases at the same rate as target peptides, quantitative linearity will always appear to be more accurate than in a real background matrix. Matrix-matched calibration curves are more effective at assessing linearity in real-world scenarios since the background signal does not change with dilution. To accomplish this, we had to build a suitable background matrix of similar composition to our target T cell proteome. Our approach used dimethyl labeling to modify the foreground T cell proteome, which kept the same composition while also producing different precursor and fragment masses. Dimethyl labeling was first introduced as a multiplexing method where multiple samples would be labeled and mixed prior to mass spectrometry.^42^ In our approach, only the background is modified, where free amines are mass-shifted by two methyl groups (28 Da). This shifts any labeled precursors (even incomplete reactions with a single methyl group) outside of the precursor isolation window used by PRM measurements, ensuring that any foreground signals will not be confused with background signals. Additionally, dimethyl labeling is affordable, easy, and quick, as peptides are labeled to 99.9% completion within a 1-hour reaction.

**Figure 3:**
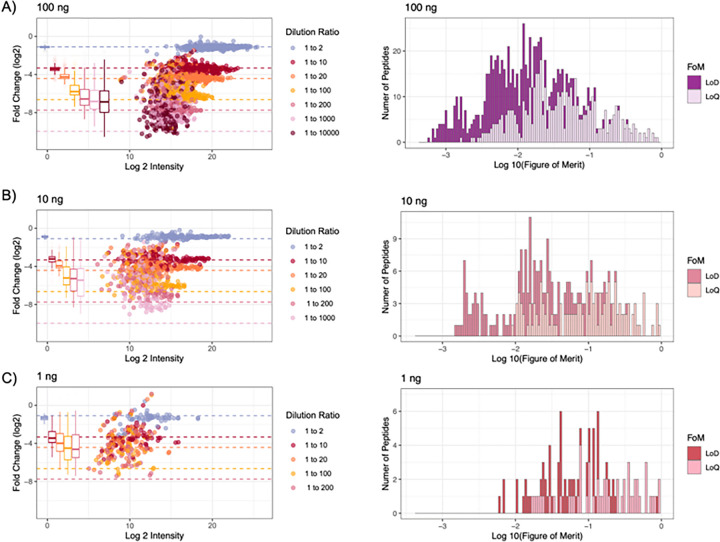
**A**) The quantitative accuracy of matrix-matched curves on an ion trap of pooled IL-2 and IL-15 peptides in a background of dimethyl-labeled pooled peptides. We generated three curves loading 100 ng, 10 ng, and 1 ng of material on-column. Each dilution is a different color where colored dashed lines indicate the expected fold change. Box plots show the spread of measured values where the whiskers indicate 5% and 95% points, and the bold line indicates the median measurement. **B**) For each curve, there is a histogram of the number of peptides with assigned lower limits of detection (LoD) and quantification (LoQ).

While ion traps generally have a more limited dynamic range compared to Orbitrap-based mass spectrometers, the sensitivity of ion traps allows for superior detection and quantification of low-input samples.^17^ Additionally, Orbitraps have slower scanning speeds compared to the Q-LIT, limiting the number of peptides that can be targeted within a cycle. Here, we found that reasonable quantitative accuracy can be achieved with the Q-LIT at low input while targeting a similar number of peptides as with higher-input Orbitrap-based PRM assays. At 100 ng, the quantitative accuracy of most peptides acquired with PRM remains consistent for nearly two orders of magnitude (Figure 3A), where the median lower limit of detection (LoD) was 0.83:100 and the median lower limit of quantification (LoQ) was 2.8:100 (Figure 3B), where only 0.6% of peptides could not be assigned a LoQ. Quantification was slightly worse at the 10 and 1 ng levels, where 4.6% and 20% of peptides could not be assigned a LoQ. Unsurprisingly, at the 100 ng level signal is more easily distinguishable from noise and the LoD distribution is generally higher than at 10 ng or 1 ng (**Supplementary Data**).

**Figure 4:**
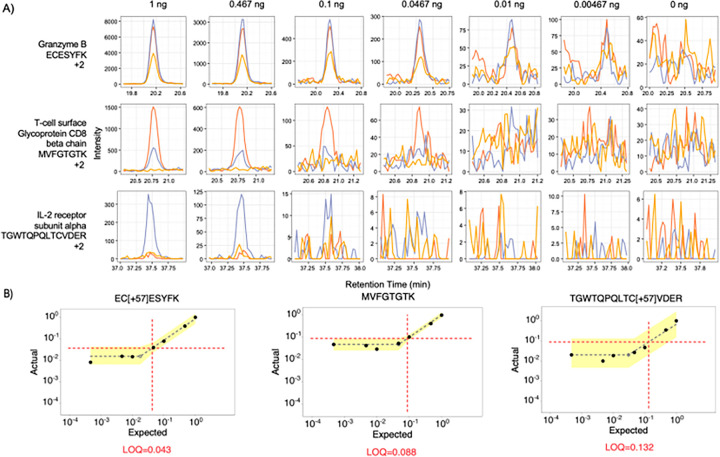
Three representative peptides that were quantifiable below 1 ng. **A**) Each row displays a peptide chromatogram at each dilution within the 1 ng curve. Each peptide contains three representative transitions. The first peptide from Granzyme B had the best estimated LoQ at 0.043:1, while the third peptide from IL-2 receptor subunit alpha had an estimated LoQ at 0.132:1 at 1 ng. **B**) LoQ and LoD were estimated on a peptide-by-peptide basis.

Single cells typically produce between 0.1 and 0.3 ng of peptides, depending on the cell type. Considering the 1 ng sample, the median measured peptide produced a linear signal in this range (0.198:1). Several peptides showed a linear response below 0.1 ng. For example, the peptide ECESYFK from Granzyme B was found to have a LoQ of 0.043:1, equating to a proteome fraction consisting of 43 pg in a background of 1 ng, and was still measurable above background at the 18 pg level (Figure 4A and 4B). Two other Granzyme B peptides, VAAGIVSYGYK and TQQVIPMVK, produced even lower LoDs (below 10 pg equivalents). Granzyme B is a serine protease implicated in multiple autoimmune diseases.^43^ All told, 61 peptides with estimated LoQs in the 1 ng assay corresponded to 30 quantified proteins. At least 6 points across the peak were sampled for all peptides in the assay achieving 8–10 points across the peak on average.

### Validated cell populations for quantitative testing

In addition to showing quantitative accuracy in a controlled matched matrix, we wanted to validate measurement precision in low-input biological experiments. The interleukins (IL) family of proteins is a class of cytokines expressed by many cells, including immune cells, which bind to specific receptors that elicit pro- and anti-inflammatory roles.^44^ Certain cytokines, such as IL-2 and IL-15, bind to receptors on the surface of T cells in specific biological events, such as activation and differentiation. Both of these molecules have been successfully used as part of immunotherapies to combat cancer.^45–48^ Interestingly, IL-2 and IL-15 are structurally similar in homology and activate T cells through the same receptor subunits (IL-2/IL-15Rβγ),^49,50^ mediating largely similar biological effects on T cells.^51,52^ However, possibly related to expression of the private IL-2Rα and IL-15Rα chains, IL-2 induces an effector-like phenotype (with low CD62L expression) while IL-15 induces a memory-like phenotype (with higher CD62L).^53^ We generated activated T cells cultured in IL-2 or IL-15 to replicate an effector-like and memory-like phenotype for CD4^+^ and CD8^+^ cells (**Supplemental Figure S2A**). We selected this model system to showcase the ability to generate LIT-PRM assays using well-studied biology at inputs below 1 ng. Additionally, flow cytometry was used as an orthogonal technique to validate the cell populations present in IL-2 and IL-15 treated T cells on days 5, 6, and 10 exhibited an effector-like and memory-like phenotype (**Supplemental Figure S2B and S2C**).

**Figure 5:**
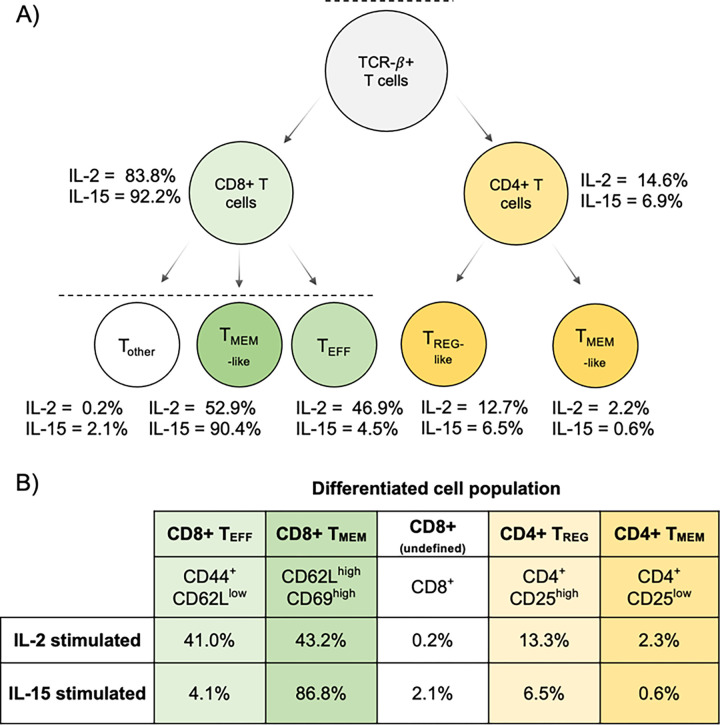
A summary of the cell populations in IL-2 and IL-15 stimulated T cells determined by the flow cytometry panel described in **Supplemental Table S1**. **A**) The gating procedure used for determining the relative percentage of each cell type in the IL-2 and IL-15 samples (more details in **Supplemental Figure S2**). **B**) The estimated cell populations based on back calculations of the gating results.

**Figure 6:**
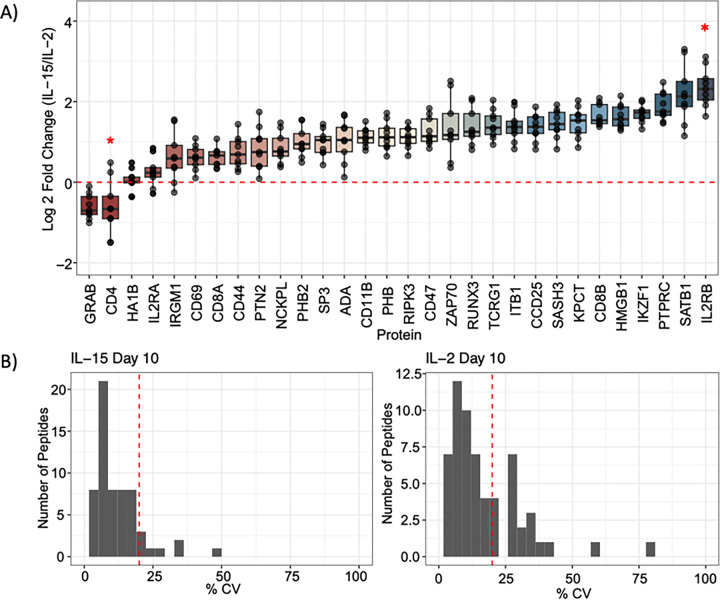
Quantifying immune cell biological replicates at 1 ng. **A**) Quantitative ratios for the panel proteins assayed in the 10 peptide/cycle PRM. The assay was collected in technical triplicate injections of Day 10 IL-2 and IL-15-stimulated T cell proteomes. The selected panel of proteins is associated with T cell activation, differentiation, or cytokine signaling. No LoQ was determined for CD4 with the 1 ng calibration curve, indicated by a red 5-point star. In the IL-2 stimulated sample, IL2RB was measured below the LoQ determined by the 1 ng calibration curve, indicated by a 6-point star. **B**) Coefficient of technical variation (% CV) plots for all peptides quantified in the 1 ng assay.

At day 10, flow cytometry identified that each culture was predominantly composed of T cells, with CD8^+^ T cells being the majority subset in both IL-2 (83.8%) and IL-15 (92.2%) cultures (Figure 5). Correspondingly, we found that CD4^+^ T cells composed 14.6% of the cells stimulated with IL-2 and 6.9% of cells stimulated by IL-15. This was reflected in our targeted proteomics data, as the CD4 protein was the second most downregulated protein in IL-15-stimulated T cells compared to IL-2-stimulated cells (Figure 6A). We note that this protein was not technically quantified at 1 ng, as the one peptide for CD4 (VVQVVAPETGLWQCLLSEGDKVK) lacked linearity in signal as estimated by the calibration curve. We measured the same peptide at the 10 ng level, where we calculated the LoD to be 0.96:10 (ratio of foreground to background) with an LoQ of 8.3:10 (**Supplemental Figure S3**). This indicated that at the 1 ng level, CD4 should be above the LoD but below the LoQ, and our results match these calculations.

IL-2 stimulation is known to push activated T cells into an effector-like population, reflected by the paired flow cytometry data on day 10. Granzyme B, from which we estimated the most responsive peptide (TQQVIPMVK) was quantitative to 0.025:1 (ratio of foreground to background), is an effector molecule secreted by cytotoxic CD8^+^ T cells. We found peptides associated with this protein were 1.55x lower in IL-15 than IL-2 stimulated cells using targeted proteomics (Figure 6A), which matches flow cytometry data indicating that the number of effector CD8^+^ T cells (T_EFF_) are lower when stimulated with IL-15 than IL-2. While both IL-2 and IL-15 resulted in the activation of T cells, IL-15 stimulation led to the differentiation of memory-like T cells, as demonstrated in the flow cytometry data. The CD44 receptor antigen is a cell surface receptor that helps cells facilitate cell-cell interaction and response to the tissue microenvironment. Interestingly, we found that the expression of CD44 is slightly higher in IL-2 compared to IL-15, indicating that IL-2 stimulated cells had a higher population of activated cells, with a 1.6x median fold change in abundance. T cells that express CD62L have an increased population of memory T cells after IL-15 stimulation.^50^ Flow cytometry data indicated that we had a higher population of CD62L^+^ cells in the IL-15 stimulated condition compared to T cells stimulated with IL-2 (**Supplemental Figure S2**), indicating a higher population of memory-like T cells. Compared to the IL-15 stimulated cells, IL-2 stimulated T cells expressed IL-2Rβ/IL-15Rβ at a higher ratio (Figure 6A), which is associated with a memory phenotype. In general, we observe high analytical precision using PRM with a Q-LIT platform, even in 1 ng assays. Most peptides were measured with less than a 20% coefficient of variation between 3 technical replicates (Figure 6B).

Ultimately, we detected 100% of the proteins monitored with flow cytometry using global proteomics during library generation. While some of these proteins were hard to observe at low input (1 ng), we were able to quantify 75% above an estimated LoQ with targeted proteomics. This overlap indiates complementary benefits of using flow cytometry in tandem with targeted proteomics to fully capture immune cell state. While single-cell proteomics using mass spectrometry continues to develop, flow cytometry is the best method for measuring a small number of proteins (6–12) on thousands of individual cells within a single day. On the other hand, targeted mass spectrometry on 1–10 T cells (equivalent to around 0.1 and 1 ng) can monitor tens to hundreds of proteins, including cytokines and transcription factors, which cannot be easily monitored using flow cytometry.

## Conclusion

Here, we demonstrate a complete workflow for acquiring global libraries and rapidly building *de novo* PRM assays using only a Q-LIT mass spectrometer. While the Q-LIT is capable of DDA, we found that equivalently large libraries could be quickly generated using GPF-DIA. We also found that PRM assays using the Q-LIT were linearly quantitative even at low input, enabling us to accurately measure difficult to measure cytokines, transcription factors, and immune proteins. From a broader perspective, high-resolution mass spectrometry is expensive in terms of instrument costs and requiring greater technical experience to operate successfully. In contrast, Q-LIT mass analyzers are easier to maintain and more cost-effective to operate in part because they have less stringent vacuum requirements compared to Orbitrap-based mass spectrometers, making them an appealing option for low-input proteomics. These factors are especially important in single-cell proteomics, where each biological sample needs thousands of injections. Our results suggest that Q-LITs provide laboratories with competent low-input proteomics analysis in situations where high resolution is impractical. We believe these instruments offer a high value-to-expense ratio, potentially democratizing mass spectrometry on a broader array of laboratory settings. This democratization is particularly impactful in immuno-oncology, where proteome-based analysis of immune cell populations can uncover crucial biomarkers to guide clinical decisions.

## Experimental Methods

### T cell cultures

Splenocytes from C57BL/6 mice were stimulated with plate-bound anti-CD3 mAb (145–2C11 clone) on day 0 in complete media, as described previously.^54^ On day 2, cells were washed and re-plated with human (h) IL-2 or hIL-15 at 200 ng/mL. Cells were washed and split on days 4 and 6. Flow cytometry was performed on days 5, 6, and 10, and cells were washed three times with DPBS, centrifuged at 500 RCF for 5 minutes, pelleted, and stored at −80°C on days 6 and 10 for mass spectrometry. A third condition was maintained without stimulation as a control for flow cytometry as well.

### Flow cytometry

Flow cytometry was performed as previously described.^54^ Briefly, cells collected on days 5, 6, and 10 were stained with live/dead fixable blue dead-cell stain (Invitrogen #L23105), and antibodies for B220, CD4, CD8, CD25, CD44, CD62L, CD69, and TCRb (see antibody details in **Supplemental Table 1**). Stained cells were acquired with a Cytek Biosciences Aurora^™^ 5-laser flow cytometer and analyzed using BD Biosciences FlowJo^™^ software.

### Proteomics sample preparation

Frozen cell pellets were lysed in a 5% SDS buffer with 50 mM TEAB, 1x HALT, and 2 mM MgCl_2_. DNA was sheared with a Bioruptor^®^ Pico by sonicating at 14°C for 30 seconds, followed by 30 seconds of rest, a total of 10 times. Sheared cells were then spun down at 13,000 RCF for 10 minutes, and the protein supernatant was retained. Protein quantities were estimated using a Pierce^™^ bicinchoninic acid (BCA) Protein Assay Kit. Proteins were reduced with 40 mM dithiothreitol (DTT), alkylated with 40 mM iodoacetamide, and quenched with 20 mM DTT. Acidification was done with 2.5% phosphoric acid, and protein was loaded onto suspension trap (s-trap) micros (Protifi LLC). Digestion was performed with trypsin at a 1:20 ratio of enzyme to protein at 47°C for 2 hours, then eluted. Peptides were dried down and stored at −80°C.

According to the kit protocol, an aliquot of dried peptides was separated according to basicity using a Pierce High pH Reverse-Phase Fractionation Kit. Briefly, 50 μg of peptides were resuspended in 0.1% trifluoroacetic acid in HPLC-grade water. The separation mini-columns from the kit were centrifuged at 5000 RCF for 2 minutes to remove any liquid and pack the resin. The mini-columns were then equilibrated with 100% acetonitrile and washed 3 times with water. Resuspended peptides were loaded, and the flow through was collected as the first fraction. The mini-columns were washed with water, and the eluent was collected as the second fraction. The elution buffers specified from the kit were then used to produce the following 8 fractions. Fractionated peptides were then dried down and stored at −80°C until mass spectrometry-based analysis for DDA-based library generation.

A separate aliquot of the eluted peptides was dimethyl labeled using an in-solution amine-labeling reaction published by Boresema et al.^42^ Digested peptides were resuspended in 100 mM TEAB (pH = 8.5). Formaldehyde (4%) was added to the resuspended peptides and mixed. Sodium cyanoborohydride (0.6 M) was then added to catalyze the dimethyl labeling reaction for 90 minutes at 22°C while mixing vigorously. The reaction was quenched with 1% ammonia and 5% formic acid. All peptides were resuspended in 2% acetonitrile with 0.1% formic acid. Calibration curves were generated by mixing labeled and unlabeled peptides at different concentrations. In these mixtures, unlabeled peptides were diluted in a dimethyl-labeled background over 4 orders of magnitude (**Supplemental Table 2**) and aliquoted at different concentrations prior to mass spectrometry analysis.

### LC-MS settings

Data was acquired on a Thermo Scientific^™^ Stellar^™^ MS coupled to a Vanquish^™^ Neo UHPLC system. Solvent A consisted of 100% water with 0.1% formic acid, and solvent B contained 80% acetonitrile with 0.1% formic acid. An Easy-Spray^™^ source was used for ionization at 2000 V, and the ion transfer tube was set to 275°C. Peptides were separated on a 25 cm C18 analytical Easy-Spray column, packed with 2 μm beads along a 50-minute linear gradient as follows: from 0–4 minutes, 2% B, 4–8 minutes increased to 8% B, 8 to 58 minutes increased with 28% B, 58 to 65 minutes increased to 44% B, followed by a 10-minute wash at 100% B. The flow for the entire gradient was set to 250 nL/min. The instrument was configured to expect chromatography of approximately 15 seconds and fragment peptides with a default charge state of 2 and a collision cell gas pressure of 8 mTorr.

### DDA on an ion trap instrument

For DDA experiments, the RF lens was set to 30%. Precursor spectra were collected ranging from 350–1250 *m/z* at a scan rate of 67 kDA/s. The automatic gain control (AGC) target was set to “Standard” with an absolute AGC target of 3e4. The maximum ion injection time (maxIIT) was set to 100 ms and spectra were collected using centroiding in positive mode. MS2 scans were collected only on peptides with a charge state greater than 1, excluding undetermined charge states. An intensity threshold of 5E2 was used to trigger an MS2 scanand an HCD NCE of 30%. Following the MS2 measurement, the peptide m/zs were placed on a dynamic exclusion list for fragmentation for 2 seconds using a precursor mass tolerance of +/− 0.5 *m/z*. Twenty DDA scans were taken in each cycle with a 1.6 m/z isolation window around the precursor of interest. Fragment ions were scanned at 125 kDa/second scan rate from 200–1500 *m/*z using an AGC target of 1E4 and a maxIIT of 50 ms.

### DIA and PRM on an ion trap instrument

For both DIA and PRM experiments, the precursor range was set to 350–1250 *m/z* and measured at a rate of 67 kDa/second. The AGC target was set to “Standard,” which is equivalent to 1e4, and the maxIIT was set to 100 ms. The loop control was set to “all.” Peptides were fragmented with HCD with NCE set to 30%, and fragments were scanned at 67 kDa/second over a range of 200–1500 *m/z*. Precursor isolation windows for DIA were consistently 8 *m/z* wide, where margins were set to forbidden zone locations. Six gas phase fractions were used to collect chromatogram libraries over 400–500, 500–600, 600–700, 700–800, 800–900 and 900–1000 *m/z*. The majority of settings were the same as a wide-window DIA scan, with the exception of 2 *m/z* wide isolation windows over the adjusted precursor *m/z* range for each method.

All settings for PRM scans were the same except for the isolation window width and maxIIT. For PRM assays at 10, 20, and 50 peptides per cycle, the maxIIT was set to 200, 95, and 50 ms, respectively. Precursor isolation windows were set to 2 *m/z*,where MS/MS were collected over 200–1500 *m/z*.

### Data analysis

Global data was first converted to the universal mzML format using peak picking. DIA data was analyzed with EncyclopeDIA v.3.0.0-SNAPSHOT using the Ion Trap/Ion Trap mode. Mass tolerance was set to 0.4 Da where a minimum of 3, but a maximum of 5 ions were used for quantification. The chromatogram library was generated by searching 6 gas phase fractions against a Prosit^38^ predicted library. The Prosit library contained spectrum predictions of all +2 and +3 ions from a mouse FASTA from UniProt, which was accessed on October 22, 2019. The predicted library allowed for up to 1 missed cleavage, with a default charge state of 3, and default NCE of 33 over 396.4–1002.7 *m/z*. Wide-window (8 *m/z*) injections were searched against the chromatogram library using the same search settings. Global DDA data was searched in Scribe using the Ion Trap/Ion Trap instrument mode for b and y tryptic peptides, with a library mass tolerance of 0.4 Da. The 10 high-pH fractionated injections were searched against the same Prosit predicted library used to generate the DIA library.

Global DIA data was searched using CHIMERYS^55^ intelligent search algorithm (MSAID GmbH) in Thermo Scientific^™^ Proteome Discoverer^™^ 3.1, using analogous settings for the EncyclopeDIA search. A predicted spectrum library was generated from the mouse fasta database by INFERYS^™^ deep learning framework (MSAID GmbH) for all tryptic +2, +3, and +4 peptides between 7–30 amino acids in length. For processing, spectrum files were selected using the ion trap MS setting, with a signal-to-noise peak threshold of 1.5. The top 24 peaks were selected in each window with a fragment mass tolerance set to 0.4 Da. Fixed carbamidomethyl modifications and a maximum of 2 oxidized methionines were allowed per peptide. The retention times from CHIMERYS were extracted for all detections and combined with EncyclopeDIA’s detections. For peptides detected by both software tools, the EncyclopeDIA retention times were preferred. The combined detections and retention times were used to select peaks within EncyclopeDIA and run against a 1% FDR to obtain a combined search engine library. The fractionated injections were also searched in Proteome Discoverer, using a mass tolerance of 0.4 Da for all +2, +3, and +4 peptides, and the same settings used for the CHIMERYS search, Skyline^56,57^ version 23.1.0.455 was used for targeted analysis. For analyzing the calibration curves and other PRM injections of IL-2 and IL-15 replicates, the chromatogram library was first imported to serve as a reference point for integrating low-input PRMs. With the imported DIA results, transition settings were altered, and the PRM samples were imported. For both imports, the settings peptide settings were set to Trypsin [KR\P], with a maximum of 2 missed cleavages, and the mouse fasta used to generate the Prosit library was used to generate a background proteome. Retention time window predictions were set to 5 minutes; however, measured retention times were used when present. Peptides between 7 and 40 amino acids in length were used, and “auto-select all matching peptides” was left checked. Only carbamidomethylation modifications were considered for cysteine. For transition settings, peptides of +2, +3, and +4 precursor charges, along with +1 and +2 fragment ion charges were considered for b and y ion types. For product ion selection, we considered the third ion to the second to last ion. DIA precursor windows were used for exclusion when importing the chromatogram library. The ion match tolerance for the library was set to 0.4 Da, and 6–9 product ions were used from filtered product ions. For the instrument parameters, a 200–1500 *m/z* range was considered, with a method match tolerance of 0.4 Da, and “dynamic min product *m/z*” and “triggered chromatogram acquisition” were checked. For the full-scan parameters, DIA was used when importing the chromatogram library file as a reference point for integrating calibration curves. The gas-phase fractionated isolation windowing scheme was imported from the files for a QIT mass analyzer, with a resolution of 0.4 Da and retention time filtering within 5 minutes of MS/MS IDs. For importing PRM injections, the “PRM” acquisition method was used rather than the DIA method. Finally, lower limits of detection (LoD) and quantification (LoQ) were estimated using EncyclopeDIA, and calculated quantities of IL-2 and IL-15 replicates were determined using calibration curves.

## Data Availability

Proteomics data and Skyline documents are available on Panorama at https://panoramaweb.org/StellarIonTrapForLowInput.url. All proteomics raw data is also publicly available on the MASSIVE repository under the accession number MSV000094904 (ftp://massive.ucsd.edu/v08/MSV000094904/). Open-source software developed for this project is publicly available as part of the EncyclopeDIA project at https://bitbucket.org/searleb/encyclopedia.

## References

[1] Kitano H. Systems biology: a brief overview. Science 295, 1662–1664 (2002). 11872829 10.1126/science.1069492

[2] Arsenio J. Early specification of CD8+ T lymphocyte fates during adaptive immunity revealed by single-cell gene-expression analyses. Nat. Immunol.15, 365–372 (2014). 24584088 10.1038/ni.2842 PMC3968536

[3] Cano-Gamez E. Single-cell transcriptomics identifies an effectorness gradient shaping the response of CD4+ T cells to cytokines. Nat. Commun.11, 1801 (2020). 32286271 10.1038/s41467-020-15543-y PMC7156481

[4] Thompson A. Tandem mass tags: a novel quantification strategy for comparative analysis of complex protein mixtures by MS/MS. Anal. Chem.75, 1895–1904 (2003). 12713048 10.1021/ac0262560

[5] Leduc A., Huffman R. G., Cantlon J., Khan S. & Slavov N. Exploring functional protein covariation across single cells using nPOP. Genome Biol.23, 261 (2022). 36527135 10.1186/s13059-022-02817-5 PMC9756690

[6] Budnik B., Levy E., Harmange G. & Slavov N. SCoPE-MS: mass spectrometry of single mammalian cells quantifies proteome heterogeneity during cell differentiation. Genome Biol.19, 161 (2018). 30343672 10.1186/s13059-018-1547-5 PMC6196420

[7] Siyal A. A. Sample Size-Comparable Spectral Library Enhances Data-Independent Acquisition-Based Proteome Coverage of Low-Input Cells. Anal. Chem.93, 17003–17011 (2021). 34904835 10.1021/acs.analchem.1c03477

[8] Derks J. Increasing the throughput of sensitive proteomics by plexDIA. Nat. Biotechnol.41, 50–59 (2023). 35835881 10.1038/s41587-022-01389-w PMC9839897

[9] Wang Y. Optimized data-independent acquisition approach for proteomic analysis at single-cell level. Clin. Proteomics 19, 24 (2022). 35810282 10.1186/s12014-022-09359-9 PMC9270744

[10] Yost R. A. & Enke C. G. Selected ion fragmentation with a tandem quadrupole mass spectrometer. J. Am. Chem. Soc.100, 2274–2275 (1978).

[11] Lange V., Picotti P., Domon B. & Aebersold R. Selected reaction monitoring for quantitative proteomics: a tutorial. Mol. Syst. Biol.4, 222 (2008). 18854821 10.1038/msb.2008.61 PMC2583086

[12] Peterson A. C., Russell J. D., Bailey D. J., Westphall M. S. & Coon J. J. Parallel reaction monitoring for high resolution and high mass accuracy quantitative, targeted proteomics. Mol. Cell. Proteomics 11, 1475–1488 (2012). 22865924 10.1074/mcp.O112.020131 PMC3494192

[13] Meng S. Proteomics Analysis of Plasma-Derived Exosomes Unveils the Aberrant Complement and Coagulation Cascades in Dermatomyositis/Polymyositis. J. Proteome Res.22, 123–137 (2023). 36507906 10.1021/acs.jproteome.2c00532 PMC9830643

[14] Hinsinger G. CD138 as a Specific CSF Biomarker of Multiple Sclerosis. Neurol Neuroimmunol Neuroinflamm 11, e200230 (2024). 38669615 10.1212/NXI.0000000000200230 PMC11057439

[15] Wu Z. Targeted Mass Spectrometry Analyses of Somatic Mutations in Colorectal Cancer Specimens Using Differential Ion Mobility. J. Proteome Res.23, 644–652 (2024). 38153093 10.1021/acs.jproteome.3c00444

[16] Senko M. W. Novel parallelized quadrupole/linear ion trap/Orbitrap tribrid mass spectrometer improving proteome coverage and peptide identification rates. Anal. Chem.85, 11710–11714 (2013). 24251866 10.1021/ac403115c

[17] Heil L. R., Remes P. M. & MacCoss M. J. Comparison of Unit Resolution Versus High-Resolution Accurate Mass for Parallel Reaction Monitoring. J. Proteome Res.20, 4435–4442 (2021). 34319745 10.1021/acs.jproteome.1c00377

[18] Schwartz J. C., Senko M. W. & Syka J. E. P. A two-dimensional quadrupole ion trap mass spectrometer. J. Am. Soc. Mass Spectrom.13, 659–669 (2002). 12056566 10.1016/S1044-0305(02)00384-7

[19] Canterbury J. D., Merrihew G. E., MacCoss M. J., Goodlett D. R. & Shaffer S. A. Comparison of data acquisition strategies on quadrupole ion trap instrumentation for shotgun proteomics. J. Am. Soc. Mass Spectrom.25, 2048–2059 (2014). 25261218 10.1007/s13361-014-0981-1 PMC4417682

[20] Borràs E., Pastor O. & Sabidó E. Use of Linear Ion Traps in Data-Independent Acquisition Methods Benefits Low-Input Proteomics. Anal. Chem.93, 11649–11653 (2021). 34404205 10.1021/acs.analchem.1c01885

[21] Phlairaharn T. High Sensitivity Limited Material Proteomics Empowered by Data-Independent Acquisition on Linear Ion Traps. J. Proteome Res.21, 2815–2826 (2022). 36287219 10.1021/acs.jproteome.2c00376

[22] Phlairaharn T. Optimizing Linear Ion-Trap Data-Independent Acquisition toward Single-Cell Proteomics. Anal. Chem.95, 9881–9891 (2023). 37338819 10.1021/acs.analchem.3c00842

[23] Searle B. C. Chromatogram libraries improve peptide detection and quantification by data independent acquisition mass spectrometry. Nat. Commun.9, 5128 (2018). 30510204 10.1038/s41467-018-07454-w PMC6277451

[24] Ma’ayan A. & Duan Q. A blueprint of cell identity. Nat. Biotechnol.32, 1007–1008 (2014). 25299921 10.1038/nbt.3035 PMC4274604

[25] Subramanian A. A Next Generation Connectivity Map: L1000 Platform and the First 1,000,000 Profiles. Cell 171, 1437–1452.e17 (2017). 29195078 10.1016/j.cell.2017.10.049 PMC5990023

[26] Michalski A. Mass spectrometry-based proteomics using Q Exactive, a high-performance benchtop quadrupole Orbitrap mass spectrometer. Mol. Cell. Proteomics 10, M111.011015 (2011). 10.1074/mcp.M111.011015 PMC3284220 21642640

[27] Scheltema R. A. The Q Exactive HF, a Benchtop mass spectrometer with a pre-filter, high-performance quadrupole and an ultra-high-field Orbitrap analyzer. Mol. Cell. Proteomics 13, 3698–3708 (2014). 25360005 10.1074/mcp.M114.043489 PMC4256516

[28] Heil L. R. Evaluating the Performance of the Astral Mass Analyzer for Quantitative Proteomics Using Data-Independent Acquisition. J. Proteome Res.22, 3290–3300 (2023). 37683181 10.1021/acs.jproteome.3c00357 PMC10563156

[29] Second T. P. Dual-pressure linear ion trap mass spectrometer improving the analysis of complex protein mixtures. Anal. Chem.81, 7757–7765 (2009). 19689114 10.1021/ac901278y PMC2810160

[30] Olsen J. V. A dual pressure linear ion trap Orbitrap instrument with very high sequencing speed. Mol. Cell. Proteomics 8, 2759–2769 (2009). 19828875 10.1074/mcp.M900375-MCP200 PMC2816009

[31] Desiere F. The PeptideAtlas project. Nucleic Acids Res.34, D655–8 (2006). 16381952 10.1093/nar/gkj040 PMC1347403

[32] Wu P. Integration and analysis of CPTAC proteomics data in the context of cancer genomics in the CBIOPortal. Mol. Cell. Proteomics 18, 1893–1898 (2019). 31308250 10.1074/mcp.TIR119.001673 PMC6731080

[33] Sharma V. Panorama Public: A Public Repository for Quantitative Data Sets Processed in Skyline. Mol. Cell. Proteomics 17, 1239–1244 (2018). 29487113 10.1074/mcp.RA117.000543 PMC5986241

[34] Ting Y. S. Peptide-Centric Proteome Analysis: An Alternative Strategy for the Analysis of Tandem Mass Spectrometry Data. Mol. Cell. Proteomics 14, 2301–2307 (2015). 26217018 10.1074/mcp.O114.047035 PMC4563716

[35] Searle B. C., Egertson J. D., Bollinger J. G., Stergachis A. B. & MacCoss M. J. Using Data Independent Acquisition (DIA) to Model High-responding Peptides for Targeted Proteomics Experiments. Mol. Cell. Proteomics 14, 2331–2340 (2015). 26100116 10.1074/mcp.M115.051300 PMC4563719

[36] Lawrence R. T., Searle B. C., Llovet A. & Villén J. Plug-and-play analysis of the human phosphoproteome by targeted high-resolution mass spectrometry. Nat. Methods 13, 431–434 (2016). 27018578 10.1038/nmeth.3811 PMC5915315

[37] Zolg D. P. INFERYS rescoring: Boosting peptide identifications and scoring confidence of database search results. Rapid Commun. Mass Spectrom. e9128 (2021) doi:10.1002/rcm.9128. 34015160

[38] Gessulat S. Prosit: proteome-wide prediction of peptide tandem mass spectra by deep learning. Nat. Methods 16, 509–518 (2019). 31133760 10.1038/s41592-019-0426-7

[39] Searle B. C. Generating high quality libraries for DIA MS with empirically corrected peptide predictions. Nat. Commun.11, 1548 (2020). 32214105 10.1038/s41467-020-15346-1 PMC7096433

[40] Thomas P. D. PANTHER: Making genome-scale phylogenetics accessible to all. Protein Sci.31, 8–22 (2022). 34717010 10.1002/pro.4218 PMC8740835

[41] Pino L. K. Matrix-Matched Calibration Curves for Assessing Analytical Figures of Merit in Quantitative Proteomics. J. Proteome Res.19, 1147–1153 (2020). 32037841 10.1021/acs.jproteome.9b00666 PMC7175947

[42] Boersema P. J., Raijmakers R., Lemeer S., Mohammed S. & Heck A. J. R. Multiplex peptide stable isotope dimethyl labeling for quantitative proteomics. Nat. Protoc.4, 484–494 (2009). 19300442 10.1038/nprot.2009.21

[43] Hiroyasu S. Granzyme B inhibition reduces disease severity in autoimmune blistering diseases. Nat. Commun.12, 302 (2021). 33436591 10.1038/s41467-020-20604-3 PMC7804321

[44] Akdis M. Interleukins, from 1 to 37, and interferon-γ: receptors, functions, and roles in diseases. J. Allergy Clin. Immunol.127, 701–21.e1–70 (2011). 21377040 10.1016/j.jaci.2010.11.050

[45] Fyfe G. Results of treatment of 255 patients with metastatic renal cell carcinoma who received high-dose recombinant interleukin-2 therapy. J. Clin. Oncol.13, 688–696 (1995). 7884429 10.1200/JCO.1995.13.3.688

[46] Rosenberg S. A. IL-2: the first effective immunotherapy for human cancer. J. Immunol.192, 5451–5458 (2014). 24907378 10.4049/jimmunol.1490019 PMC6293462

[47] Rubinstein M. P. Phase I Trial Characterizing the Pharmacokinetic Profile of N-803, a Chimeric IL-15 Superagonist, in Healthy Volunteers. J. Immunol.208, 1362–1370 (2022). 35228263 10.4049/jimmunol.2100066

[48] Mullard A. First-in-class IL-15 receptor agonist nabs FDA approval for bladder cancer. Nat. Rev. Drug Discov. (2024) doi:10.1038/d41573-024-00073-9. 38671056

[49] Kennedy M. K. Reversible Defects in Natural Killer and Memory Cd8 T Cell Lineages in Interleukin 15–Deficient Mice. J. Exp. Med.191, 771–780 (2000). 10704459 10.1084/jem.191.5.771 PMC2195858

[50] Kanegane H. & Tosato G. Activation of naive and memory T cells by interleukin-15. Blood 88, 230–235 (1996). 8704178

[51] Arneja A., Johnson H., Gabrovsek L., Lauffenburger D. A. & White F. M. Qualitatively different T cell phenotypic responses to IL-2 versus IL-15 are unified by identical dependences on receptor signal strength and duration. J. Immunol.192, 123–135 (2014). 24298013 10.4049/jimmunol.1302291 PMC3950894

[52] Ring A. M. Mechanistic and structural insight into the functional dichotomy between IL-2 and IL-15. Nat. Immunol.13, 1187–1195 (2012). 23104097 10.1038/ni.2449 PMC3501574

[53] Castro I., Yu A., Dee M. J. & Malek T. The basis of distinctive IL-2– and IL-15–dependent signaling: Weak CD122-dependent signaling favors CD8+ T central-memory cell survival but not T effector-memory cell development. J. Immunol.187, 5170–5182 (2011). 21984699 10.4049/jimmunol.1003961 PMC3304468

[54] Andrijauskaite K. IL-12 conditioning improves retrovirally mediated transduction efficiency of CD8+ T cells. Cancer Gene Ther.22, 360–367 (2015). 26182912 10.1038/cgt.2015.28 PMC4807400

[55] Frejno M. Unifying the analysis of bottom-up proteomics data with CHIMERYS. BioRxiv 2024.05.27.596040 (2024) doi:10.1101/2024.05.27.596040. PMC12074992 40263583

[56] MacLean B. Skyline: an open source document editor for creating and analyzing targeted proteomics experiments. Bioinformatics 26, 966–968 (2010). 20147306 10.1093/bioinformatics/btq054 PMC2844992

[57] Pino L. K. The Skyline ecosystem: Informatics for quantitative mass spectrometry proteomics. Mass Spectrom. Rev.39, 229–244 (2020). 28691345 10.1002/mas.21540 PMC5799042

